# Overexpression of the Receptor for Advanced Glycation Endproducts (RAGE) Is Associated with Poor Prognosis in Gastric Cancer

**DOI:** 10.1371/journal.pone.0122697

**Published:** 2015-04-10

**Authors:** Da Wang, Tingting Li, Gengtai Ye, Zhiyong Shen, Yanfeng Hu, Tingyu Mou, Jiang Yu, Sihao Li, Hao Liu, Guoxin Li

**Affiliations:** 1 Department of General Surgery, Nanfang Hospital, Southern Medical University, Guangzhou, Guangdong, P.R. China; 2 International Department, the Affiliated High School of SCNU, Guangzhou, Guangdong, P.R. China; IRCCS National Cancer Institute, ITALY

## Abstract

**Background:**

The receptor for advanced glycation endproducts (RAGE) is an oncogenic multidisciplinary trans-membranous receptor, which is overexpressed in multiple human cancers. Recently, it has been shown that RAGE is also involved in carcinogenesis and tumor invasion. In this study, we investigated the expression levels and prognostic value of RAGE in primary gastric cancers (GC).

**Methods:**

We investigated RAGE expression in primary GC and paired normal gastric tissue by real-time quantitative RT-PCR (n = 30) and Western blotting analysis (n = 30). Additionally, we performed immunohistochemistry on 180 paraffin-embedded GC specimens, 69 matched normal specimens.

**Results:**

RAGE was overexpressed in GC compared with the adjacent noncancerous tissues (*P*＜0.001), and higher RAGE expression significantly correlated with the histological grade (*P* = 0.002), nodal status(*P* = 0.025), metastasis status(*P* = 0.002), and American Joint Committee on Cancer stage (*P* = 0.020). Furthermore, upregulation of RAGE expression is an independent prognostic factor in multivariate analysis using the Cox regression model (*P* = 0.001).

**Conclusions:**

RAGE Overexpression may be a useful marker to predict GC progression and poor prognosis.

## Introduction

The receptor for advanced glycation end products (RAGE), a cell surface molecule expressed in a range of cell types, is a multiligand member of the immunoglobulin superfamily [1]. RAGE has been implicated in a wide spectrum of pathological responses, including inflammation and cancer, based on the variety of its ligands [2]. As a newly recognized oncogenic trans-membranous receptor, it has been shown to be involved in multiple human cancers stimulating growth, survival, and metastatic spread [3]. Several studies have demonstrated that RAGE is upregulated and loses its characteristic granular pattern in primary tumors [4]. The AGE-RAGE interaction plays a crucial part in the development of prostate cancer, and inhibiting this interaction has potential as a new molecular target for cancer prevention or therapy [5]. Co-expression of RAGE and High-mobility group protein B1 (HMGB1) has been substantially associated with colorectal cancer invasion and metastasis [4,6,7]. Additionally, RAGE expression correlates with the angiogenesis and tumor metastasis in oral squamous cell carcinoma (OSCC) and may be an independent prognostic factor for recurrence and prognosis in OSCC patients [8,9]. Furthermore, genetic polymorphism of RAGE gene can be a predictive biomarker used to screen for patients sensitive to thermotherapy and to evaluate the prognosis of non-small cell lung cancer (NSCLC) patients [10].

However, the role of RAGE in gastric cancer (GC)has remained elusive. RAGE has previously been indicated closely associated with invasion and metastasis in gastric cancer [11]. Moreover, it was reported that blockade of the RAGE signaling suppressed the growth and metastases of gastric cancer cells [12]. In an investigation into the roles of RAGE in GC pathogenesis and progression and the effects of its gene polymorphisms on receptor function, Gu and colleagues [13] have demonstrated that RAGE Gly^82^Ser polymorphism may be associated with an increased risk of initiation or progression of gastric carcinoma. However, to our knowledge, the relationship between RAGE expression and GC patient prognosis is unknown.

In this study, we analyzed RAGE expression level in GC by using real-time quantitative RT-PCR (qRT-PCR), Western blotting and immunohistochemistry (IHC). We identified a relationship between RAGE expression and the clinicopathological features of GC, and then evaluated the prognostic value of RAGE expression for the postoperative survival of GC patients.

## Materials and Methods

### Ethics statement

This study was approved by the Nanfang Hospital Ethical Committee and written informed consent was obtained by all these patients involved.

### Patients and tissue specimens

Thirty paired fresh-frozen gastric cancerous and corresponding noncancerous mucosa tissue (excised > 10 cm away from the edge of the GC) samples were collected from GC patients within 30 min after resection and immediately stored in liquid nitrogen until use. Another 180 paraffin embedded GC tissue blocks and 69 control samples from matched normal gastric tissues taken from the distal resection margin were obtained from the stored files of the Department of General Surgery, Nangfang Hospital, Southern Medical University, were collected surgically between January 2007 and December 2008. The 180 patients included 120 men and 60 women aged 22–78 years (mean, 55.62 years). None of these patients were treated with radiotherapy or chemotherapy prior to surgery. The histopathological type and stage of the resected specimens were determined according to the criteria of the World Health Organization classification and the American Joint Committee on Cancer (AJCC) Cancer Staging Manual (the 7th edition, 2010). Among them, 125 Stage I–III patients received radical resection (19, 25 and 81 for Stages I, II and III, respectively), and 55 Stage-IV patients received palliative surgery and/or chemotherapy. The metastasis-affected distant organs in Stage-IV patients included the peritoneum, distant lymph node, liver, transverse colon, ovary or oviduct, pancreas, intestine, bone, lung, brain and inferior vena cava. The patients were followed until death or the last follow-up date (30 December 2013). The median follow-up time was 48 months (from 2 to 82 months).

### Total RNA extraction and real-time quantitative RT-PCR

Total RNA was extracted from minced Tissues with the TRIzol reagent (TaKaRa, Japan) and reverse transcribed to first-strand cDNA with the TaqMan Reverse Transcription Kit (TaKaRa, Japan). Then, 0.5- to 1-μl aliquots of the cDNA were used as templates to amplify the RAGE fragment (forward: 5- AAACATCACAGCCCGGATTG-3; reverse: 5- TCCGGCCTGTGTTCAGTTTCT-3) under the following conditions: 95°C for 30 s; 40 cycles of 95°C for 5s; 60°C for 30 s; and melting at 95°C for 5 sec, followed by 60°C for 1 min, and cooling at 50°C for 30 sec. PCR and data collection were performed on LightCycler480 System(Roche, Swiss) with 2×SYBR Green master mix kit (Invitrogen, Carlsbad, California, USA). RAGE expression was normalized to that of GAPDH.

### Western blotting analysis

Western blotting was performed according to standard methods as described previously [14]. Briefly, tissues were ground and lysed with RIPA lysis buffer and the lysates were harvested by centrifugation (12,000 rpm) at 4°C for 30 min. Protein concentrations were determined using the Bicinchoninic Acid Protein Assay Kit (Pierce, Rockford, IL, USA). Approximately 50μg samples was then separated electrophoretically on 10% sodium dodecyl sulfate (SDS)-polyacrylamide gels and transferred to a polyvinylidene difluoride membrane. After blocking the non-specific binding sites, the membrane was incubated with a rabbit polyclonal anti-RAGE antibody (Santa-Cruz, CA, USA) at a 1:1,000 dilution at 4°C overnight. After three washes with TBST (tris-buffered saline with tween-20) for 10 min, the membranes were incubated with a secondary antibody at a dilution of 1:3000 at room temperature for 60 min. Proteins were detected with an enhanced chemiluminescence system (Amersham Pharmacia Biotechnology, Piscataway, NJ, USA), and normalized to β-actin detected using a mouse anti-human β-actin antibody (1:1000 dilution; Sigma, St. Louis, MO, USA).

### Immunohistochemical assay

The IHC assay was performed as previously described [12,14]. Briefly, the slides were deparaffinized with dimethylbenzene and rehydrated through an ethanol gradient (100%, 95%, 90%, 80% and 70% ethanol) into water. After washing with PBS (phosphate-buffered saline) for three times, slides were boiled in antigen retrieval buffer, 0.01 M sodium citrate-hydrochloric acid (pH = 6.0), for 30 min in a microwave oven. After endogenous peroxidase activity was quenched with 3% H_2_O_2_, subsequent to three PBS washes, nonspecific antibody binding was blocked by incubating the slides with 10% normal goat nonimmune serum. The sections were then incubated at 4°C overnight with the rabbit polyclonal RAGE antibody (Santa-Cruz, CA, USA) at a 1:400 dilution and subsequently incubated with horseradish peroxidase (HRP) (ChemMateTM DAKO EnVisionTM Detection Kit) at room temperature for 30 min. After washing in PBS, the sections were then developed using 3,3-V-diaminobenzidine (Sigma), washed in running tap water, and lightly counterstained with hematoxylin before dehydration and coverslip mounting. Negative control experiments were conducted by replacing the primary antibody with PBS.

Immunostaining was analyzed by two independent observers (Y.X. and L.Z.) who were blinded to the patients’ outcome and other clinicopathological parameters. The RAGE detection system has been described previously [12]. All samples were categorized by the extent of immunoreactivity: 0, < 5%; 1, 5%-10%; 2, 10%–50%; 3, 50%–75%; 4, >75%. Staining intensity was scored as 0, negative; 1, weak; 2, moderate; 3, strong. For each case, the total immunostaining score also known as the staining index (SI), was calculated by multiplying the percentage of positive cells with the staining intensity score, yielding a value between 0 and 12. For this study, an optimal cutoff value was identified as follows: SI<8 was used to indicate low RAGE expression and SI≥8 high RAGE expression.

### Statistical analysis

Statistical analysis was performed using the SPSS statistical software package (SPSS version 19.0; SPSS, Chicago, IL, USA). A paired-samples t-test was used to compare the RAGE mRNA and protein levels in the cancerous and adjacent noncancerous tissue samples. The Mann–Whitney U test or Pearson Chi-Square test was used to compare the SI scores obtained by immunohistochemical analysis of the GC and corresponding normal mucosa tissue. The relationship between RAGE expression and clinicopathological characteristics were analyzed using the Pearson Chi-square test. Survival curves were calculated according to the Kaplan–Meier method and analyzed using the log-rank test. The Cox proportional hazards regression model was used for univariate and multivariate analysis to assess the hazard ratio (HR) and identify factors that predict survival. A two-sided p-value <0.05 were considered statistically significant.

## Results

### Overexpression of RAGE in human GC

**Fig 1:**
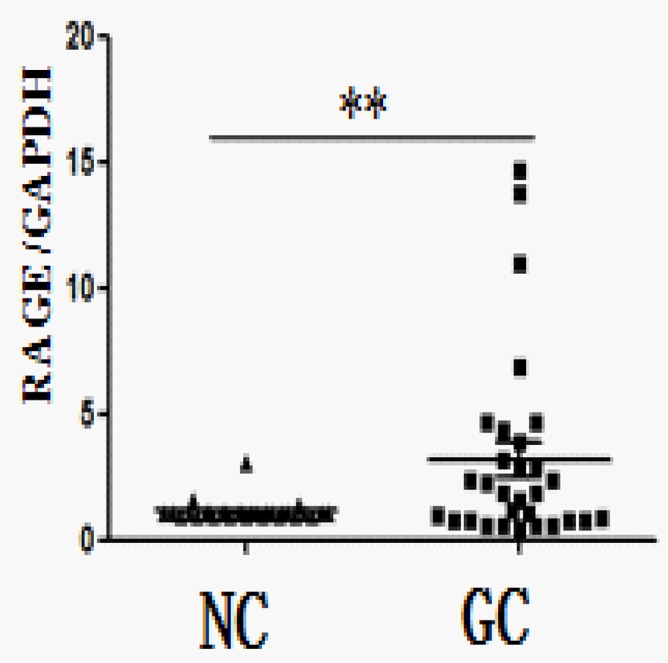
Overexpression of RAGE in gastric cancer as detected by qRT-PCR. Relative elevated expression of RAGE in gastric carcinoma tissues compared to adjacent non-cancerous tissues assessed by qRT-PCR. **P = 0.0057(pared t test, n = 30). GC, gastric carcinoma tissues; NC, adjacent non-cancerous tissues.

**Fig 2:**
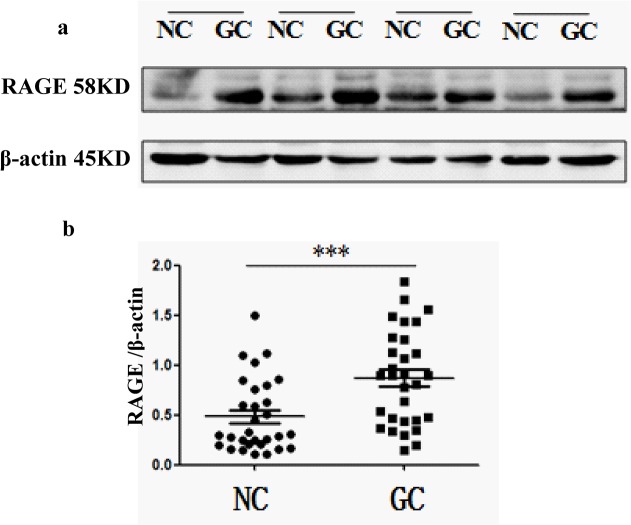
Overexpression of RAGE in gastric cancer as detected by Westen blotting analysis. **a.** Data presented here are representative of all the samples. **b.** Relative expression of RAGE was significantly increased in gastric carcinoma tissues compared to adjacent non-cancerous tissues assessed by Westen blotting. *** *P*<0.001(pared t test, n = 30). GC, gastric carcinoma tissues; NC, adjacent non-cancerous tissues.

**Fig 3:**
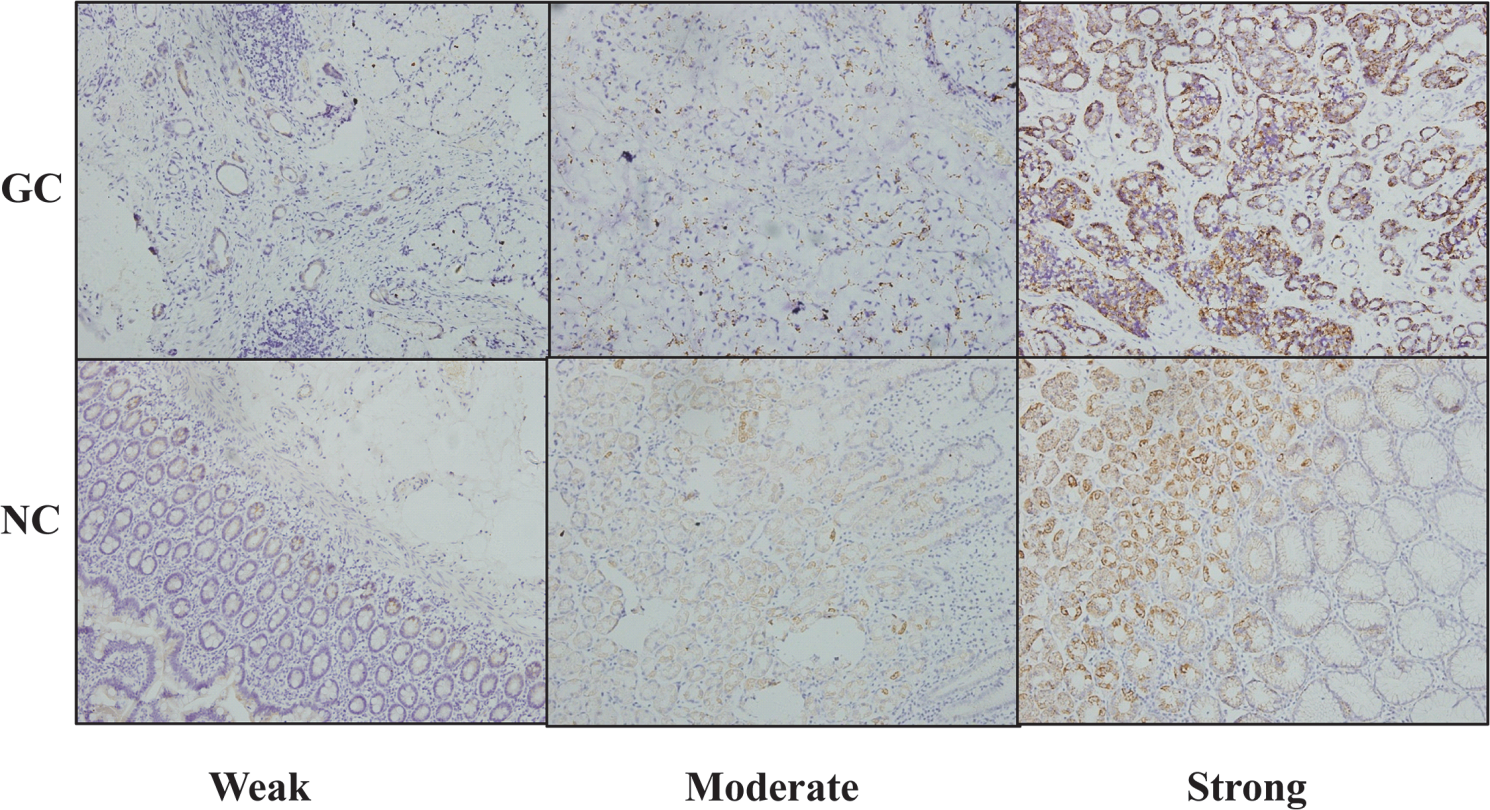
Immunochemistry(IHC) analysis of RAGE expression in gastric carcinoma tissues(GC) and adjacent non-cancerous tissues(NC); ×200.

To elucidate the role of RAGE in GC development and progression, we first analyzed its expression in GC samples and matched adjacent noncancerous tissues at the mRNA level. Real-time quantitative RT-PCR analysis showed that RAGE was overexpressed in the majority of GC tissues compared with their normal counterparts (19/30; 63.3%) (Fig 1). RAGE was similarly upregulated at the protein level in GC, as shown by Western blot and IHC assays. Elevated RAGE expression level was found by the Western blotting analysis in 26 of 30 (86.7%) GCs compared with normal tissues (Fig 2). Paraffin-embedded blocks (n = 249) from 180 GC patients were evaluated for RAGE protein expression by IHC (Table 1). Absent or low staining (SI<8) of normal gastric mucosa was observed in 55 of 69 cases (79.7%), whereas high staining (SI≥8) was noted in the majority of GC tissues(111/180; 61.7%). RAGE expression was significantly higher in GC tissues (n = 180; SI = 6.22) compared with normal gastric tissues (n = 69; SI = 2.09; *P*<0.001). Representative examples of weak, moderate, strong staining (micrographs with 200× magnification) are shown in Fig 3.

**Table 1:** Immunohistochemical score of the RAGE protein expression in gastric cancer and adjacent noncancerous tissues.

	NC(%)	GC(%)	***p***
SI(mean±SD)	2.09±3.526	6.22±5.082	<0.001
SI<8	55(79.7%)	69(38.3%)	<0.001
SI≥8	14(20.3%)	111(61.7%)	

GC, gastric carcinoma tissues; NC, adjacent non-cancerous tissues; SI, the staining index.

### Association of RAGE expression with clinicopathological characteristics

Based on the categories that we defined in the the Methods section, the data showed that the high expression of RAGE was significantly correlated with histological grade (*P* = 0.002), nodal status (N stage, *P* = 0.025), metastasis status (M stage, *P* = 0.002) and AJCC stage (*P* = 0.020), but was not with gender, age, tumor size, tumor location, Lauren classification or tumor invasion (Table 2).

**Table 2:** Relationship between RAGE expression and clinicopathologic features of patients with gastric cancer.

Variable	No. of patients	RAGE expression		*p* value
		Low(%)	High(%)	
Gender				0.515
Male	120	48(40.0)	72(60.0)	
Female	60	21(35.0)	39(65.0)	
Age (Years)				0.453
<60	72	30(41.7)	42(58.3)	
≥60	108	39(36.1)	69(63.9)	
Tumor Size(cm)*				0.095
<4	74	23(33.3)	51(45.9)	
≥4	106	46(66.7)	60(54.1)	
Tumor Location				0.934
Cardia	38	17(24.6)	21(18.9)	
Body	37	12(17.4)	25(22.5)	
Antrum	98	37(53.6)	61(55.0)	
Whole	7	3(4.3)	4(3.6)	
Histological Grade				**0.002**
Well	18	13(18.8)	5(4.5)	
Moderate	53	23(33.3)	30(27)	
Poor And Undifferentiated	109	33(47.8)	76(68.5)	
Lauren Classification				0.432
Intestinal Type	29	13	16	
Diffuse Type	151	56	95	
Tumor Invasion				0.530
T1+T2	25	11(15.9)	14(12.6)	
T3+T4	155	58(84.1)	97(87.4)	
Nodal Status				**0.025**
Absent(N0)	46	24(34.8)	22(19.8)	
Present(N1-3)	134	45(65.2)	89(80.2)	
Metastasis Status				**0.002**
Absent(M0)	127	58(84.1)	69(62.2)	
Present(M1)	53	11(15.9)	42(37.8)	
AJCC Stage				**0.020**
I	19	10	9	
II	25	12	13	
III	81	35	46	
IV	55	12	43	

AJCC, American Joint Committee on Cancer.

### Correlation between RAGE expression based on IHC and patient survival

**Fig 4:**
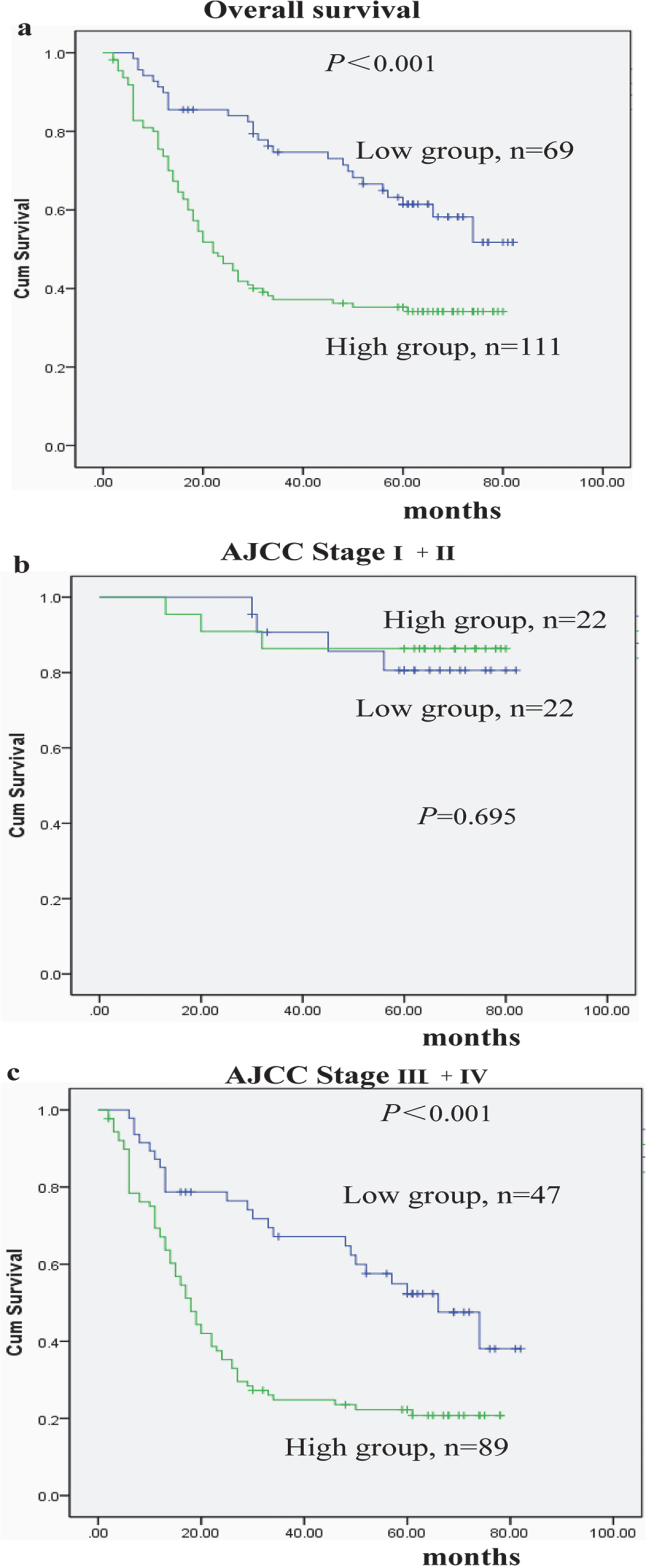
Kaplan–Meier survival curves in gastric cancer patients grouped according to RAGE expression levels. **a**. Patients with high RAGE expression had a shorter overall survival time than those with low RAGE expression (*P*<0.001). Similar findings was observed in the AJCC stage III+ IV subgroup (*P*<0.001)(**c**), but not in the AJCC stage I+II subgroup (*P* = 0.695)(**b**).

The Kaplan–Meier curve showed that patients in high-RAGE group had a significantly worse overall survival than patients in low-RAGE group (*P*<0.001; Fig 4A). Patients with high RAGE expression also had a significantly worse overall survival than those with low expression of RAGE in the AJCC stage III/ IV subgroup (n = 136;*P*<0.001; Fig 4C), but not in the AJCC stage I/II subgroup (n = 44; *P* = 0.695; Fig 4B).

### Univariate and Multivariate Analyses

Univariate analysis showed a significant relationship between overall survival and age, tumor invasion, nodal status, metastasis status, AJCC stage and RAGE expression (see Table 3). All these factors are included in a multivariate Cox proportional hazards model to adjust for the effects of the covariates. Based on this model, RAGE expression levels (hazard ratio (HR) = 2.143; 95% confidence interval (95% CI) = 1.357–3.383; *P* = 0.001) and tumor invasion (HR = 11.209; 95% CI = 1.297–96.874; *P* = 0.028) were confirmed as independent prognostic factors (Table 3).

**Table 3:** Univariate and multivariate analysis of clinicopathological parameters for correlation with overall survival.

Variables	Univariate ^a^			Multivariate ^b^		
	HR	CI(95%)	*p* value	HR	CI(95%)	*p* value
Gender(female vs male)	0.857	0.570, 1.288	0.458			
Age(Years)(≥60 vs <60)	1.692	1.138, 2.515	**0.009**	1.172	0.779, 1.762	0.447
Tumor Size(cm) (≥4vs <4)	1.453	0.959, 2.200	0.078	1.108	0.725, 1.694	0.636
Location(Cardia/Body/Antrum/Whole)	1.094	0.854, 1.400	0.476			
Differentiation Status(G3/G2/G1)	1.267	0.929, 1.728	0.135			
Lauren Classification(Diffuse/Intestinal)	1.417	0.858, 2.338	0.173			
Tumor Invasion(T3+T4/T1+T2)	25.344	3.531,181.893	**0.001**	11.209	1.297, 96.874	**0.028**
Nodal Status(N1-3/N0)	4.800	2.491, 9.248	**<0.001**	1.696	0.659, 4.362	0.273
Metastasis Status(M1/M0)	5.835	3.859, 8.823	**<0.001**	2.796	0.829, 9.424	0.097
AJCC Stage(IV/III/II/I)	3.815	2.757, 5.278	**<0.001**	1.404	0.475, 4.155	0.539
RAGE Expression(high/low)	2.314	1.484, 3.609	**<0.001**	2.143	1.357, 3.383	**0.001**

HR, hazard ratio; CI, confidence interval; AJCC, American Joint Committee on Cancer;

^a^ Hazard ratios in univariate models;

^b^ Hazard ratios in multivariable models.

## Discussion

GC remains one of the most fatal human malignancies, especially in East Asia. Despite advances in diagnosis and therapy, the prognosis for gastric cancer is still dismal and is dependent on a series of carcinoma characteristics, such as tumor growth, differentiation, invasion and distant metastasis [15]. GC tumorigenesis and progression are poorly understood, complex, multistep processes regulated by intrinsic and extrinsic cellular signals involving multiple gene and protein alterations. It is vital to identify and understand the molecular mechanisms underlying tumor initiation and progression to develop rational and cancer-specific approaches to the early diagnosis and treatment of GC.

In this study, we investigated the RAGE expression status in GC at both the mRNA and protein levels and found that RAGE was remarkably upregulated in human GC tissues compared with normal gastric epithelium. IHC analysis was performed to further validate this upregulation of RAGE expression in primary GC. We observed that a high level of RAGE expression was significantly associated with the histological grade, nodal status, metastasis status, and AJCC stage in GC, similar to previous findings in OSCC and NSCLC [9,10]. Taken together, these results suggested that RAGE may be involved in GC progression.

We also determined for the first time that RAGE expression is a strong predictor of poor prognosis for GC patients, similar to findings in colorectal cancer, hepatocellular cancer, OSCC and NSCLC [9,10,16,17]. However, the prognostic impact of RAGE expression appears to differ according to cancer stage (Fig 4). In the present study, survival analysis suggested that tumor invasion and RAGE expression independently predicted poor overall survival. The relatively small sample sizes of the stage I and II group might be responsible for the difference. Most patients with GC in China are diagnosed in in the advanced stages due to a lack of efficient early detection. Additionally, RAGE expression has a narrower 95% CI and appears to be a a better predictor for GC compared with tumor invasion in this study.

RAGE is a pattern recognition receptor that binds multiple ligands derived from a damaged cell environment, and plays a critical role in promoting the gastrointestinal tumourigenesis [18]. Also as an important inflammatory mediator, RAGE could modulate crosstalk between survival pathways and autophagy in tumor cells and promote tumor survival via sustaining autophagy and limiting apoptosis [19]. Kuniyasu *et al*. [11] have reported that, RAGE expression is closely associated with the invasion and metastasis in GC patients, which provides us an experimental basis for the functional study of RAGE in gastric cancer. Subsequently, Xu and colleagues [12] demonstrated that the knockdown of RAGE reduced GC cell proliferation and invasion of gastric cancer, decreased expression of AKT, PCNA and MMP-2, and induced cell apoptosis and cycle arrest. Therefore, targeted inhibition of RAGE or its ligands may serve as novel targets to enhance current cancer therapies.

To date, the value of targeting RAGE for possible early intervention and prophylaxis in GC remains unclear. However, several studies have revealed the role of RAGE in other cancer types and have demonstrated that the blockade of RAGE decreased growth and metastases of both implanted tumors and tumors developing spontaneously in susceptible mice. Inhibition of RAGE decreased proliferation in breast cancer cells and induced cell apoptosis and inhibited prostate cancer growth [20,21]. RAGE Dysfunction also inhibited angiogenesis and progression of colorectal cancer, and prolonged the survival in pancreatic cancer [22–24]. Blockade of RAGE with soluble RAGE (sRAGE) attenuated liver injury, thereby improving survival, protecting against hepatocellular necrosis, and enhancing the expression of proregenerative cytokine tumor necrosis factor-a [25]. Moreover, Moy et al. [26] found a borderline statistically significant inverse association between continuous sRAGE exposure and liver cancer risk, suggesting that sRAGE may protect against the inflammatory effects caused by RAGE activation. These findings consistently indicated that RAGE might also act as a potential therapeutic target in GC. Given its biological characteristics, RAGE and its derivatives have been considered as a new indicator for malignant potential including GC. The RAGE Gly^82^Ser polymorphism has been shown to be associated with an increased risk for GC development or progression in a hospital-based case-control study [13].

In the present study, we found that upregulation of RAGE expression was significantly associated with poor clinicopathological characteristics and poor overall survival, suggesting that it may contribute to the malignant potential of GC. Therefore, RAGE could therefore serve as a valuable novel biomarker for predicting prognosis and a potential therapeutic target for patients with GC. However, further studies are warranted to clarify the underlying mechanisms of RAGE overexpression, thereby contributing to better understanding and further developing its potential use.
